# Unveiling the role of oxidative stress in ANCA-associated glomerulonephritis through integrated machine learning and bioinformatics analyses

**DOI:** 10.1080/0886022X.2025.2499905

**Published:** 2025-05-14

**Authors:** Liyuan Xie, Xianying Qiu, Junya Jia, Tiekun Yan, Pengcheng Xu

**Affiliations:** Department of Nephrology, Tianjin Medical University General Hospital, Tianjin, P.R. China

**Keywords:** ANCA-associated glomerulonephritis, oxidative stress, machine learning, WGCNA, bioinformatic analysis

## Abstract

Anti-neutrophil cytoplasmic antibody (ANCA)-associated vasculitis (AAV) is a systemic autoimmune disease often leading to rapidly progressive glomerulonephritis. Oxidative stress plays a critical role in the development and progression of ANCA-associated glomerulonephritis (AAGN), but the underlying mechanisms remain poorly understood. Targeting genes related to oxidative stress may provide novel insights and supplementary therapeutic benefits for AAGN. In the current study, we obtained differentially expressed genes from AAGN-related microarray datasets in the Gene Expression Omnibus database, and oxidative stress-related genes (OSRGs) from the GeneCards and Gene Ontology databases to identify differentially expressed OSRGs. Then, by integrating weighted gene co-expression network analysis, and machine learning algorithms, we identified four upregulated hub OSRGs (all *p* < 0.01) with strong diagnostic potential (all AUC > 0.9)-CD44, ITGB2, MICB, and RAC2 – in the AAGN glomerular training dataset GSE104948 and validation dataset GSE108109, along with two hub OSRGs (all *p* < 0.05) with better diagnostic potential (all AUC > 0.7) – upregulated gene VCAM1 and downregulated gene VEGFA-in the AAGN tubulointerstitial training dataset GSE104954 and validation dataset GSE108112. The GSEA analysis suggested that these hub genes may play a role in inflammatory and immune response processes. Moreover, we constructed regulatory networks and identified drugs that potentially target these hub genes. It’s to be noted that RAC2 and ITGB2 were associated with cyclophosphamide in the AAGN glomerular compartment, while VCAM1 and VEGFA were associated with dexamethasone in the tubulointerstitial compartment. This study offers novel insights into immune-associated OSRGs within the glomerular and tubulointerstitial compartments of AAGN which may serve as innovative targets for diagnosing and treating AAGN.

## 1. Introduction

Anti-neutrophil cytoplasmic antibody (ANCA)-associated vasculitis (AAV) is a systemic autoimmune disease characterized by vascular wall inflammation, fibrinoid necrosis, and the destruction of small to medium-sized blood vessels, accompanied by circulating ANCA. The clinical symptoms of AAV are complex, primarily presenting as systemic symptoms and damage to various organ systems. Patients usually have pulmonary hemorrhage and acute kidney injury [1,2]. Renal impairment resulting from AAV is referred to as ANCA-associated glomerulonephritis (AAGN). AAGN is a form of ­oligimmune necrotizing crescent glomerulonephritis, primarily characterized by hematuria, proteinuria, and renal impairment, which can lead to rapid deterioration of renal function and respiratory failure even death [2,3]. The mechanism underlying ANCA-induced vasculitis primarily involves the mediation of the overactivation of neutrophils, leading to the release of inflammatory cytokines, reactive oxygen species, and lytic enzymes. However, the molecular mechanisms underlying the pathogenesis of AAV remain poorly understood [4,5]. Current studies indicate that immune infiltration and oxidative stress play critical roles in the onset and progression of AAGN [6,7]. In the kidney, ANCA activates neutrophils through recognition and binding to antigens present on their surface, subsequently inducing respiratory burst and degranulation, then neutrophils release reactive oxygen species and various proteases along glomerular endothelial cells, resulting in direct damage to vascular endothelial cells [8–10].

**Figure 1.**
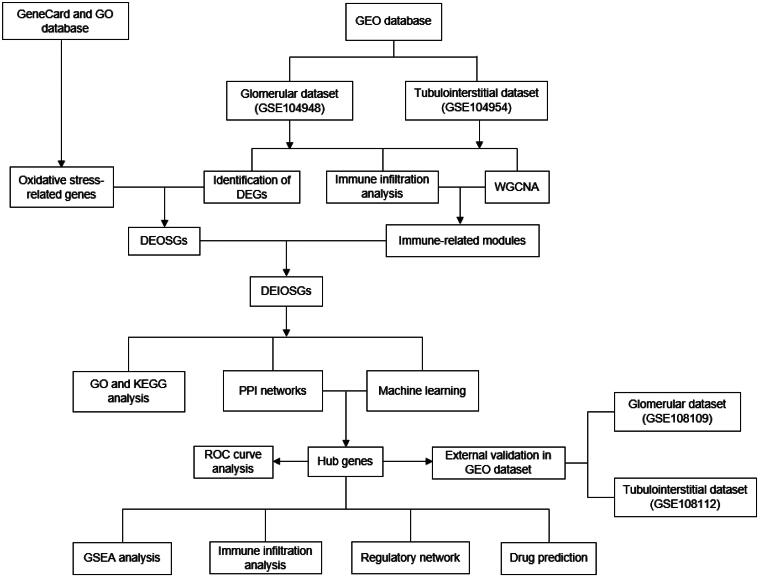
The workflow for this study. DEGs: differentially expressed genes. DEOSGs: differentially expressed oxidative stress genes; DEIOSGs: differentially expressed immune-related oxidative stress genes; GO: Gene ontology; GEO: Gene expression Omnibus; GSEA: Gene set enrichment analysis; KEGG: Kyoto Encyclopedia of genes and Genomes; PPI: protein-protein interaction; ROC: receiver operating characteristic.

In recent years, high-throughput sequencing technology has boosted bioinformatics in identifying core genes related to disease onset and progression, providing a basis for diagnosis, treatment, and new drug development [11]. The Gene Expression Omnibus (GEO) database, as a comprehensive repository, contains an extensive collection of bioinformatics datasets essential for disease-related research. Among the diverse analytical methodologies in bioinformatics, weighted gene co-expression network analysis (WGCNA) and machine learning algorithms have emerged as powerful tools for elucidating complex biological networks and patterns. To our knowledge, few studies have utilized bioinformatics analysis to characterize the oxidative stress in renal glomerular and tubulointerstitial tissue of AAV. In this research, we employed integrated machine learning and bioinformatics approaches to identify hub genes that exhibit differential expression of immune-associated oxidative stress genes within the glomerular and tubulointerstitial compartments in AAGN, potentially enhancing our understanding of the mechanisms underlying AAV, providing new insights into molecular targeted therapies and prognostic predictions. The workflow for this study is illustrated in Figure 1.

## 2. Materials and methods

### 2.1. Sources of data

The gene expression microarray datasets associated with AAGN were retrieved from the GEO database (http://www.ncbi.nlm.nih.gov/geo/), a publicly accessible functional genomics data repository containing microarray dataset, single-cell datasets, and bulk RNA-seq datasets [12]. The following keywords were used to query the GEO database: AAV or ANCA or glomerulonephritis, with the Homo sapiens filter applied. Each dataset was manually curated to select renal biopsy samples from Homo sapiens. The inclusion criteria for this study were: (1) human renal biopsy tissue studies; (2) expression data for both experimental and control groups; and (3) inclusion of patients diagnosed with AAV. The exclusion criteria included: (1) dual-channel microarray studies; (2) studies without a control group; and (3) samples from cell lines or animal models. Finally, we selected four AAGN datasets: GSE104948 and GSE108109, which contained samples from the glomerular compartment, and GSE104954 and GSE108112, which included samples from the tubulointerstitial compartment. The datasets of GSE104948 and GSE104954 are based on the GPL22945 platform, using the GeneChip Human Genome U133 Plus 2.0 array (Affymetrix; Thermo Fisher Scientific, Inc., Waltham, MA), while GSE108109 and GSE108112 are based on the GPL19983 platform using the GeneChip Human Gene 2.1 ST Array (Affymetrix; Thermo Fisher Scientific, Inc., Waltham, MA). In this study, the GSE104948 and GSE104954 datasets were used as training sets, while the GSE108109 and GSE108112 datasets served as external validation sets. We obtained 40 samples from the GSE104948 dataset, including 18 healthy controls and 22 patients with AAV. From the dataset of GSE104954, we acquired 39 samples consisting of 18 healthy controls and 21 patients with AAV. The GSE108109 dataset included 6 healthy controls and 15 patients with AAV, and the GSE108112 dataset contained 5 healthy controls and 57 patients with AAV.

### 2.2. Identification of DEGs and oxidative stress-related genes

The downloaded gene probes were converted into gene symbols. Probes without corresponding symbols or those linked to multiple genes were removed. Using the ‘limma’ package (version 3.60.6) in R (version 4.4.1), each expression matrix was normalized individually, and differentially expressed genes (DEGs) were then identified within each dataset. The DEGs between AAGN samples and normal samples in the GSE104948 and GSE104954 datasets were identified based on criteria of an adjusted *p-*value < 0.05 and |log2(fold change) | > 0.585. Additionally, heatmaps and volcano plots of the DEGs were generated using the pheatmap (version 1.0.12) and ggplot2 (version 3.5.1) R packages.

We queried ‘oxidative stress’ in the Gene Ontology (GO) database (http://geneontology.org/) using ‘Homo sapiens’ as the filter, which yielded a total of 458 oxidative stress-related genes (OSRGs). Concurrently, we retrieved OSRG from the GeneCards database (https://www.genecards.org/), applying a correlation score threshold greater than 7 as our selection criterion, which identified 1,225 OSRGs. Subsequently, after removing duplicates, we identified a total of 1,434 OSRGs for further analysis. The intersection of OSRGs selected from the database and DEGs from the GSE104948 dataset was considered to represent glomerular differentially expressed oxidative stress genes (DEOSGs) in AAGN. Similarly, the intersection with DEGs from the GSE104954 dataset was regarded as indicative of DEOSGs in tubulointerstitial compartments of AAGN. We utilized the VennDiagram (version 1.7.3) R package to visualize a Venn diagram illustrating relationships between the OSRGs and DEGs.

### 2.3. Immune infiltration analysis and weighted gene co‑expression network analysis

Cell-type identification by estimating relative subsets of RNA transcripts (CIBERSORT) uses a deconvolution algorithm to estimate the composition and abundance of immune cells in cell mixtures based on transcriptomic data [13]. In this study, CIBERSORT was used to assess the proportions of 22 immune cell types in normal and AAGN samples from GSE104948 and GSE104954. WGCNA was performed using the R package ‘WGCNA’ (version 1.73) to identify modules most relevant to immune cells in AAGN patients [14]. Initially, hierarchical clustering was conducted on research samples to detect and remove outliers. Subsequently, a correlation matrix was constructed with the WGCNA software package; an optimal soft threshold was applied to convert it into an adjacency matrix, followed by the creation of a topological overlap matrix (TOM). Based on TOM phase anisotropy measures, genes exhibiting similar expression patterns were classified into gene modules through average linkage hierarchical clustering. WGCNA then calculated correlations between these modules and differentially infiltrated immune cells. Modules with high correlation coefficients were identified as candidate modules related to differentially infiltrated immune cells for further analysis. In this study, we identified key modules with correlation coefficients between gene modules and immune cells exceeding 0.7 in absolute value for further investigation. The intersection of DEOSGs with genes in key modules was analyzed using the ‘VennDiagram’ R package, defining them as differentially expressed immune-related oxidative stress genes (DEIOSGs) for further study.

### 2.4. Functional enrichment analysis

Based on the Kyoto Encyclopedia of Genes and Genomes (KEGG) and GO databases, we used the ‘ClusterProfiler’ package in R (version 4.12.6) to perform GO and KEGG enrichment analyses on the intersecting genes [15,16]. The GO database categorizes gene functions into three categories: cellular component (CC), molecular function (MF), and biological process (BP). Additionally, KEGG enrichment analysis helps identify key signaling pathways related to gene enrichment. *p* value < 0.05 suggests that the corresponding gene set is significantly enriched.

### 2.5. Construction of protein-protein interaction network

The Search Tools for the Retrieval of Interacting Genes (STRING) (https://cn.string-db.org/) database was used to construct a protein-protein interaction (PPI) network. This PPI network was subsequently visualized using Cytoscape software (version 3.10.2). Subsequently, the maximal clique centrality (MCC) algorithm was used through the cytoHubba plug-in in Cytoscape to assess differential genes. The top 10 genes identified by this algorithm were then intersected with the top 20 genes selected by the random forest (RF) algorithm to identify hub genes.

### 2.6. Machine learning

The least absolute shrinkage and selection operator (LASSO) is a logistic regression technique that identifies significant variables and constructs an optimal classification model by applying the L1 penalty (lambda) to eliminate the coefficients of non-significant variables [17]. Support vector machine recursive feature elimination (SVM-RFE) is a supervised learning methodology designed to identify core genes by systematically eliminating feature vectors generated by SVM [18]. The random forest (RF) algorithm is a decision tree-based machine learning approach that evaluates the importance of each variable by scoring its contribution [19]. We applied these three machine learning algorithms to assess DEIOSGs from the GSE104948 and GSE104954 datasets. The genes identified by all three algorithms were then intersected and considered as hub genes.

### 2.7. Expression and ROC curve analysis

The expression levels of hub genes in AAGN and normal samples were represented through box plots generated by the R package ‘ggplot2’ (version 3.5.1). The pROC package (version 1.18.5) in R was used to calculate the area under the curve (AUC) and its 95% confidence interval (CI) to evaluate the predictive value of each selected hub gene [20]. A hub gene with an AUC > 0.7 was considered significant for disease diagnosis. Independent external datasets (GSE108109 and GSE108112) were used to validate candidate gene expression and diagnostic value. Genes that demonstrated consistent performance across datasets were designated as final hub genes. Additionally, the correlations among these hub genes were analyzed using the ‘corrplot’ package (version 0.94).

### 2.8. GSEA analysis

We conducted the single-gene Gene Set Enrichment Analysis (GSEA) to elucidate the role of hub genes. GSEA was used to identify altered pathways between the hub and biological process genomes in the GO database. It compared the expressed transcripts with corresponding entries and assessed the statistical enrichment levels of related gene sets [21].

### 2.9. Regulatory networks and target drugs

Using NetworkAnalyst (https://www.networkanalyst.ca/), we accessed the JASPAR and TarBase databases to predict transcription factors (TFs) and miRNAs for candidate genes, respectively [22]. The results were subsequently visualized using Cytoscape software. On the DGIdb website (https://dgidb.org/), small molecule drugs were queried by gene name to obtain drug-gene interaction pairs, which were then visualized as networks using Cytoscape software [23].

## 3. Results

### 3.1. Identification of DEGs

**Figure 2.**
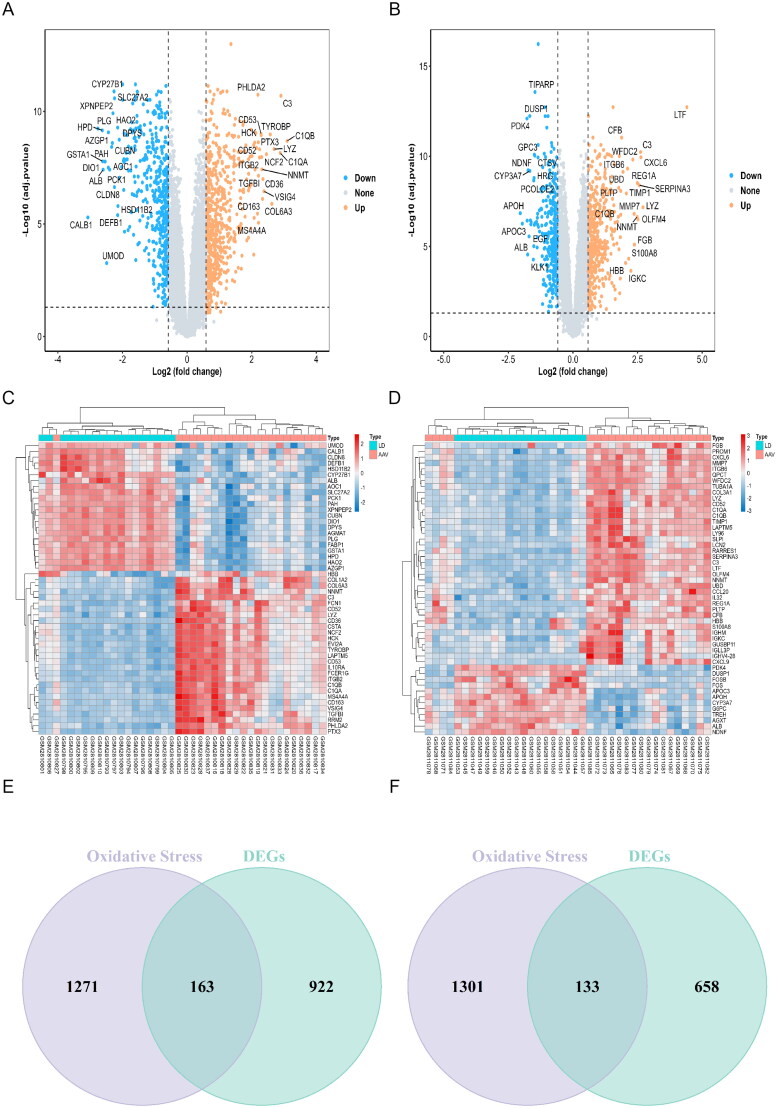
DEGs Analysis for GSE104948 and GSE104954 datasets. (A) 1085 DEGs are shown in the volcano plot of the GSE104948 dataset which contains 464 upregulated genes in orange and 621 downregulated genes in blue. The grey points represent genes with no significant difference. (B) 791 DEGs are shown in the volcano plot of the GSE104954 dataset which contains 462 upregulated genes in orange and 329 downregulated genes in blue. The grey points represent genes with no significant difference. (C) The heatmap shows the top 50 most significant DEGs in the GSE104948 dataset. Red indicates a relatively high expression and blue indicates a relatively low expression. (D) The heatmap shows the top 50 most significant DEGs in the GSE104954 dataset. Red indicates a relatively high expression and blue indicates a relatively low expression. (E) Venn diagrams of DEOSGs in the GSE104948 dataset. (F) Venn diagrams of DEOSGs in the GSE104954 dataset. DEGs were identified by the criteria of|log2fold change (FC)|>0.585 and adj. *p*. value <0.05. AAV: anti-neutrophil cytoplasmic antibody-associated vasculitis; DEGs: differentially expressed genes. DEOSGs: differentially expressed oxidative stress genes; LD: living donors.

Differential gene expression analysis of the glomerular dataset GSE104948 was conducted using |log2FC| > 0.585 and adjusted *p*-value < 0.05 as criteria. A total of 1,085 DEGs were identified, including 464 down-regulated and 621 up-regulated genes (Figure 2(A)). Similarly, differential gene expression analysis of the renal tubule dataset GSE104954 yielded 791 DEGs, including 329 down-regulated and 462 up-regulated genes (Figure 2(B)). The DEGs exhibited significantly distinct expression patterns between AAGN and normal samples (Figure 2(C,D)).

### 3.2. Identification of DEOSGs in AAGN

From the Genecard and GO databases, 1,434 OSRGs were identified. By intersecting 1,085 DEGs from the AAGN glomerular dataset GSE104948 with these OSRGs using R software, we identified 163 DEOSGs based on their shared overlap (Figure 2(E)). In the AAGN tubulointerstitial dataset, 791 DEGs were intersected with these OSRGs, resulting in 133 DEOSGs associated with the tubulointerstitial compartment after preliminary screening (Figure 2(F)).

### 3.3. Immune infiltration analysis

**Figure 3.**
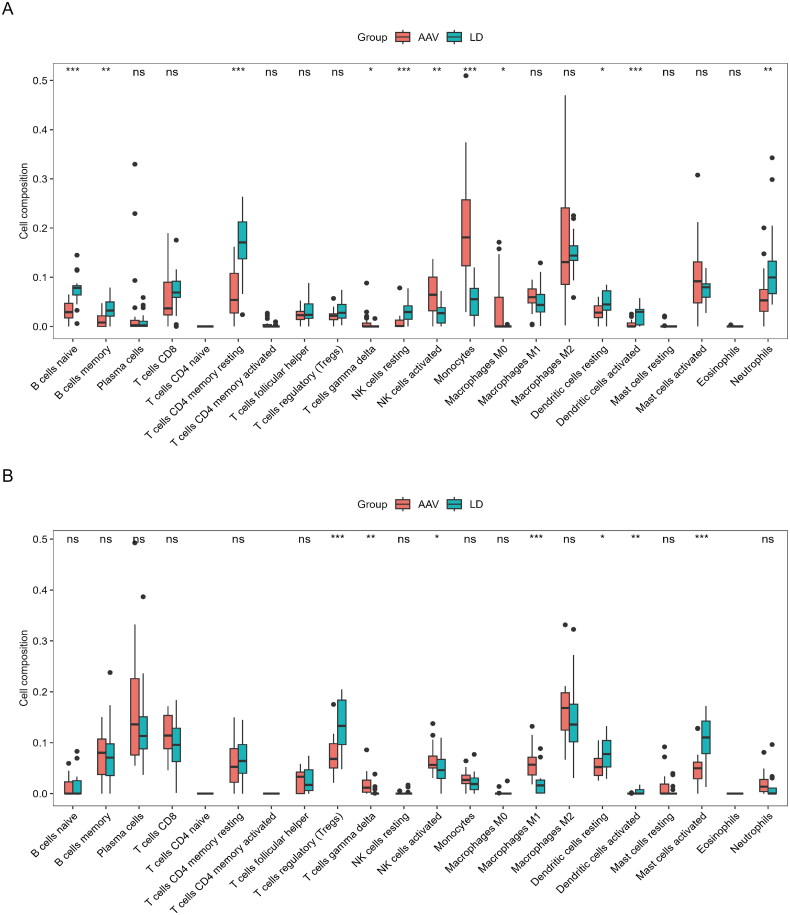
Immune infiltration analysis. (A) 22 immune cells in samples with living donors and rapidly progressive glomerulonephritis in the GSE104948 dataset. (B) 22 immune cells in samples with living donors and rapidly progressive glomerulonephritis in the GSE104954 dataset. AAV: anti-neutrophil cytoplasmic antibody-associated vasculitis; LD: living donors. The Wilcoxon rank sum was used to determine statistical significance level (α = 0.05). *p < 0.05, **p < 0.01, ***p < 0.001, ns: not significant.

We analyzed immune cell infiltration in AAGN and normal samples with the CIBERSORT algorithm. The results showed 11 comparable immune cell types in glomerular samples of AAGN and normal controls, including naive B cells, memory B cells, resting CD4 memory T cells, gamma delta (γδ) T cells, resting NK cells, activated NK cells, monocytes, M0 macrophages, resting dendritic cells, activated dendritic cells, and neutrophils (Figure 3(A)). Additionally, seven comparable immune cell types in the tubulointerstitial compartment were found in AAGN and normal controls: regulatory T cells (Tregs) cells, γδT cells, activated NK cells, M1 macrophages, resting dendritic cells, activated dendritic cells, and mast cells (Figure 3(B)).

### 3.4. Construction of weighted gene coexpression networks

**Figure 4.**
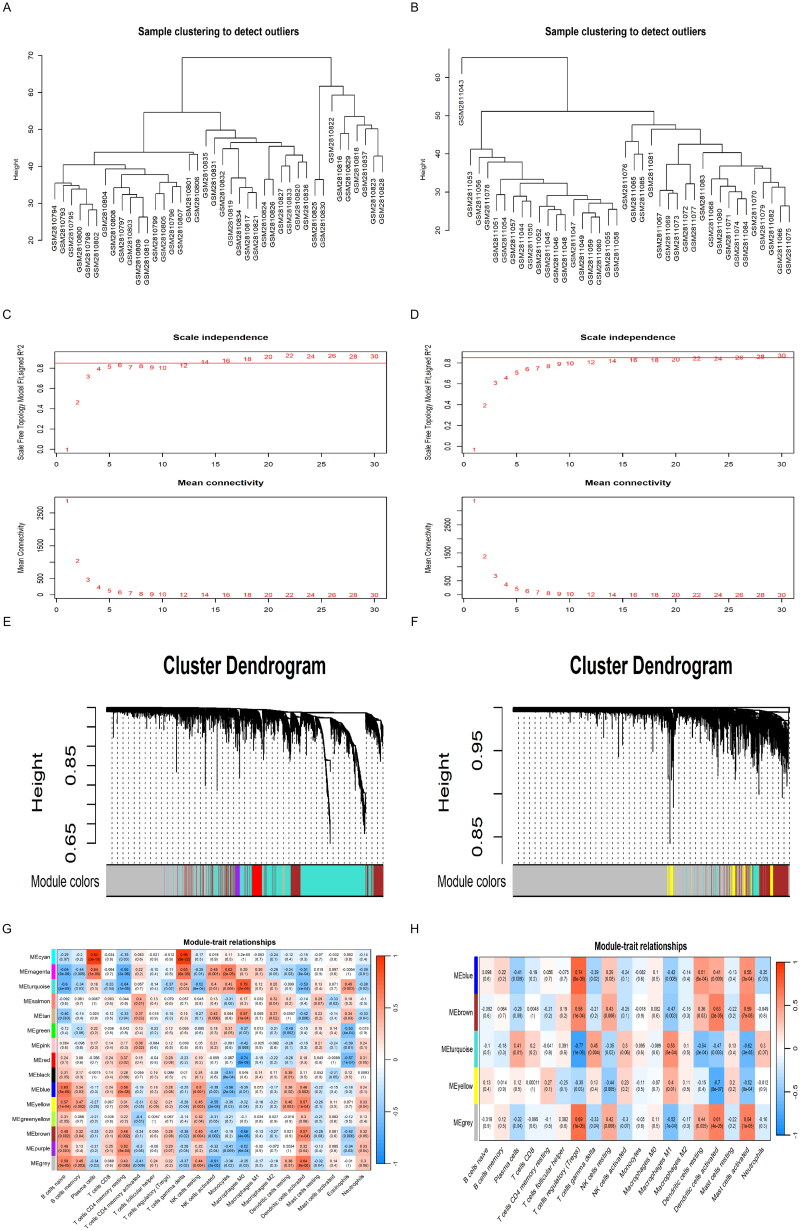
Construction of weighted gene co-expression networks analysis. (A) Outlier sample detection of GSE104948 dataset. The gene sets from all samples are contained in the dendrogram. (B) Outlier sample detection of GSE104954 dataset. The gene sets from all samples are contained in the dendrogram. (C) Soft threshold screening of GSE104948 dataset. (D) Soft threshold screening of GSE104954 dataset. (E) WGCNA modules of GSE104948 dataset. (F) WGCNA modules of GSE104954 dataset. (G) 14 modules of the GSE104948 dataset were revealed by the WGCNA. (H) 4 modules of the GSE104954 dataset were revealed by the WGCNA. WGCNA: weighted gene co-expression network analysis.

In the WGCNA, all samples from the GSE104948 and GSE104954 datasets were incorporated into the dendrogram, ensuring no outliers were present (Figure 4(A,B)). In the AAGN glomerular dataset, a soft threshold power of 14 was determined (scale-free R^2^ = 0.85) (Figure 4(C)), and subsequent WGCNA analysis revealed 14 distinct modules (Figure 4(E)). The AAGN tubulointerstitial dataset demonstrated a soft threshold power calibration of 26 (scale-free R^2^ = 0.85) (Figure 4(D)), resulting in four identified modules *via* WGCNA analysis (Figure 4(F)). Based on strict criteria (|Cor| > 0.7), hub gene modules were selected, with 2,771 genes from the cyan, turquoise, and red modules in the glomerular dataset being prioritized for further investigation. Likewise, the blue and turquoise modules in the tubulointerstitial dataset, comprising 1,789 genes, were also chosen for detailed investigation. In the glomerular dataset, the cyan module displayed a strong positive correlation with both plasma cell subsets (Cor = 0.93, *p* = 3e−18) and γδT cell subsets (Cor = 0.96, *p* = 9e−22). Meanwhile, the turquoise module showed a strong positive correlation with M0 macrophage subsets (Cor = 0.79, *p* = 2e-09), while the red module exhibited a significant negative correlation with the M0 macrophage subset (Cor= −0.74, *p* = 5e-08) (Figure 4(G)). In the tubulointerstitial dataset, the Tregs cell subset showed a strong positive correlation with the blue module (Cor= 0.74, *p* = 9e-08), whereas the turquoise module displayed a significant negative correlation (Cor=−0.77, *p* = 1e-08) (Figure 4(H)).

### 3.5. Acquisition and functional enrichment analysis of DEIOSGs

**Figure 5.**
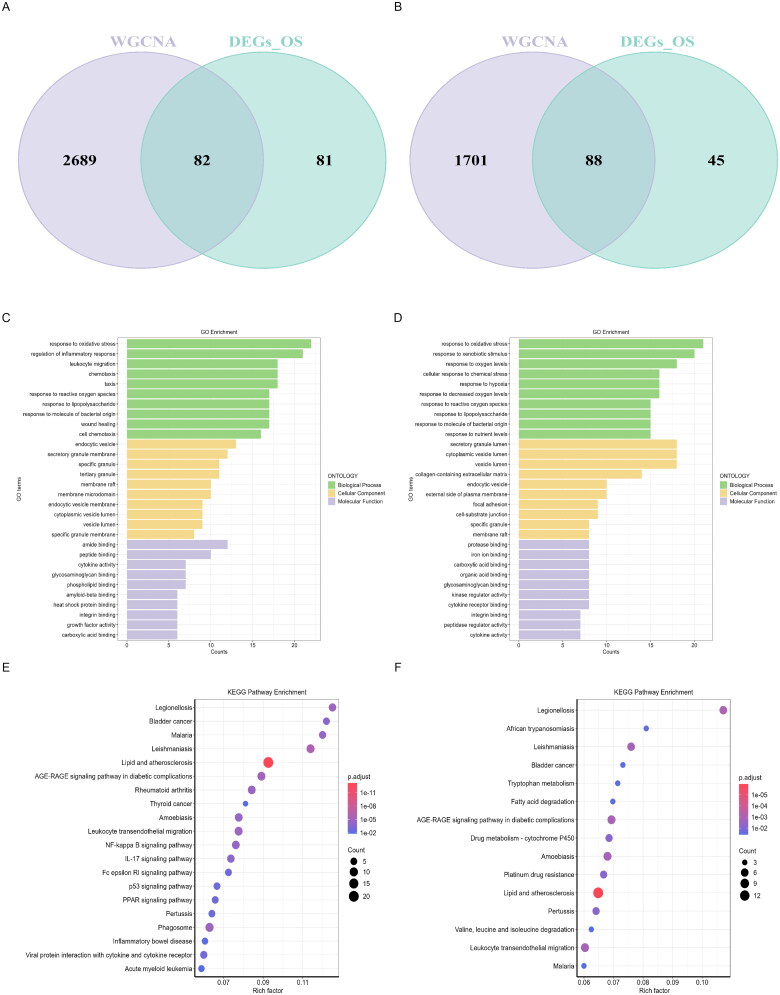
Acquisition and functional enrichment analysis of DEIOSGs. (A) Venn diagrams of DEIOSGs of GSE104948 dataset. (B) Venn diagrams of DEIOSGs of GSE104954 dataset. (C) GO enrichment analysis of the GSE104948 dataset. (D) KEGG pathway enrichment analysis of GSE104948 dataset. (E) GO enrichment analysis of the GSE104954 dataset. (F) KEGG pathway enrichment analysis of GSE104954 dataset. DEGs: differentially expressed genes; DEIOSGs: differentially expressed immune-related oxidative stress genes; GO: Gene ontology; GEO: Gene expression omnibus; KEGG: Kyoto encyclopedia of genes and genomes. OS: oxidative stress. WGCNA: Weighted gene Co-expression network analysis.

By intersecting the 163 DEOSGs in the GSE104948 dataset with the 2,771 genes from the cyan, turquoise, and red modules, we identified 82 DEIOSGs associated with AAGN glomeruli (Figure 5(A), Supplementary Table S1). By overlapping the 133 DEOSGs in the GSE104954 dataset with the 1,789 genes from the blue and turquoise modules, we identified 88 DEIOSGs specifically associated with the AAGN tubulointerstitial compartment (Figure 5(B), Supplementary Table S1). To explore the potential biological functions and signaling pathways of DEIOSGs associated with AAGN, we conducted GO and KEGG functional enrichment analyses. Notably, DEIOSGs from the glomerular region were primarily enriched in processes related to oxidative stress and inflammation (Figure 5(C)). The DEIOSGs from the tubulointerstitial compartment showed significant enrichment in processes such as oxidative stress responses, reactions to external stimuli, and adaptations to varying oxygen levels (Figure 5(D)). According to KEGG analysis, DEIOSGs from the glomerular and tubulointerstitial compartments were significantly enriched in pathways such as the AGE-RAGE signaling pathway in diabetic complications, lipid metabolism, and atherosclerosis (Figures 5(E,F)). In summary, these findings highlight the key biological processes and signaling pathway dysregulations driving the progression of AAGN.

### 3.6. Screening hub genes by machine learning and construction of PPI network

**Figure 6.**
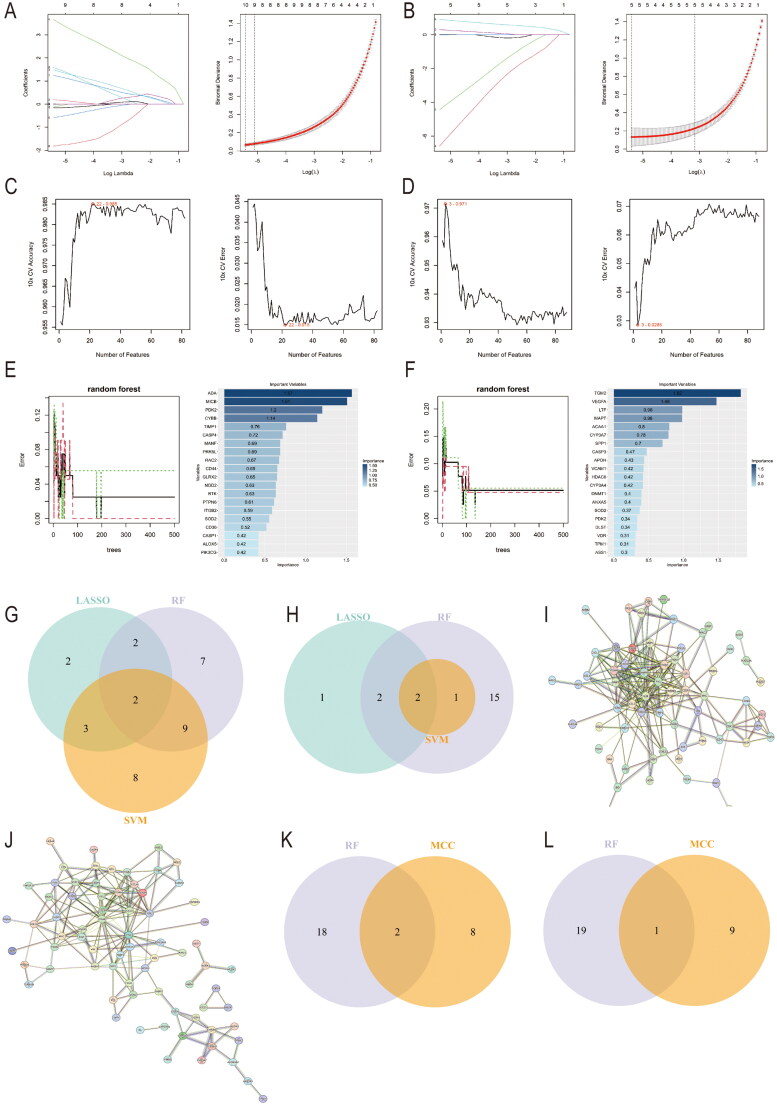
Screening hub genes by machine learning PPI network. (A) LASSO regression algorithm of DEIOSGs in GSE104948 dataset. (B) LASSO regression algorithm of DEIOSGs in GSE104954 dataset. (C) SVM-RFE algorithm of DEIOSGs in GSE104948 dataset. (D) SVM-RFE algorithm of DEIOSGs in GSE104954 dataset. (E) RF algorithm of DEIOSGs in GSE104948 dataset. (F) RF algorithm of DEIOSGs in GSE104954 dataset. (G) Venn diagrams for three algorithms in GSE104948. (H) Venn diagrams for three algorithms in the GSE104954 dataset. (I) The PPI network of DEIOSGs in the GSE104948 dataset. Interactions among 82 DEIOSGs were illustrated using the STRING online database (http://string-db.org). Network nodes represent proteins, and edges represent protein-protein associations. (J) The PPI network of DEIOSGs in the GSE104954 dataset. Interactions among 88 DEIOSGs were illustrated using the STRING online database (http://string-db.org). Network nodes represent proteins, and edges represent protein-protein associations. (K) The MCC algorithms in the cytoHubba plugin of the GSE104948 dataset. (L) The MCC algorithms in the cytoHubba plugin of the GSE104954 dataset. DEIOSGs: differentially expressed immune-related oxidative stress genes; LASSO: least absolute shrinkage and selection operator; MCC: maximal clique centrality; PPI: protein-protein interaction; RF: random Forest; SVM-RFE: support vector machine recursive feature elimination.

In the GSE104948 dataset, the LASSO regression algorithm identified 9 genes from the DEIOSGs (Figure 6(A), Supplementary Table S2). Subsequently, the SVM-RFE algorithm pinpointed 22 genes (Figure 6(C), Supplementary Table S2), and the RF algorithm selected 20 genes (Figure 6(E), Supplementary Table S2). By overlapping the results of the three methods in a Venn diagram, two candidate genes, MICB and RAC2, were identified (Figure 6(G)). For the GSE104954 dataset, 5 genes were extracted *via* the LASSO regression algorithm (Figure 6(B), Supplementary Table S2), 3 genes were identified through the SVM-RFE algorithm (Figure 6(D), Supplementary Table S2), and 20 genes were selected with the RF algorithm (Figure 6(F), Supplementary Table S2). After overlapping these results in a Venn diagram, two candidate genes, MAPT and VEGFA, were obtained (Figure 6(H)).

The PPI networks of DEIOSGs from the glomerular and tubulointerstitial compartments were presented in Figure 6(I,J), respectively. By intersecting the top ten genes from this algorithm with the twenty genes selected by the RF algorithm, two hub genes (CD44 and ITGB2) were identified in the glomerular dataset, and one candidate gene (VCAM1) was identified in the tubulointerstitial dataset (Figure 6(K,L)).

Finally, through the combination of PPI networks and machine learning algorithms, four candidate hub genes (CD44, ITGB2, MICB, and RAC2) were identified from the GSE104948 dataset, and three candidate hub genes (VCAM1, MAPT, and VEGFA) were identified from the GSE104954 dataset.

### 3.7. Expression of hub genes and the ROC curve analysis

**Figure 7.**
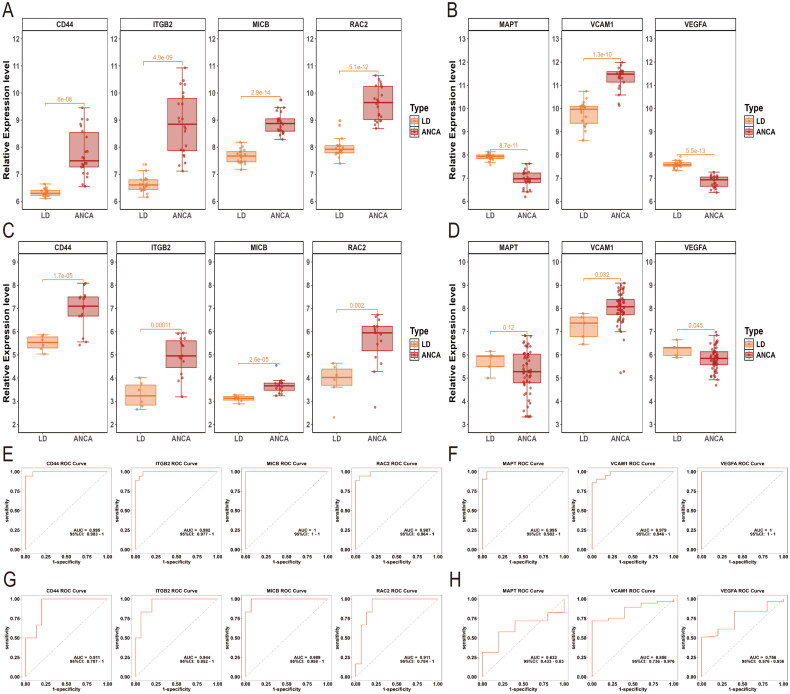
Expression of hub genes and ROC curve analysis. (A) Expression of candidate hub genes (CD44, ITGB2, MICB, and RAC2) in the GSE104948 dataset. (B) Expression of candidate hub genes (MAPT, VCAM1, and VEGFA) in the GSE104954 dataset. (C) Expression of candidate hub genes (CD44, ITGB2, MICB, and RAC2) in the GSE108109 dataset. (D) Expression of candidate hub genes (MAPT, VCAM1, and VEGFA) in the GSE108112 dataset. (E) The ROC curve analysis of candidate hub genes (CD44, ITGB2, MICB, and RAC2) in the GSE104948 dataset. (F) The ROC curve analysis of candidate hub genes (MAPT, VCAM1, and VEGFA) in the GSE104954 dataset. (G) The ROC curve analysis of candidate hub genes (CD44, ITGB2, MICB, and RAC2) in the GSE108109 dataset. (H) The ROC curve analysis of candidate hub genes (MAPT, VCAM1, and VEGFA) in the GSE108112 dataset. ANCA: anti-neutrophil cytoplasmic antibody; LD: living donors; ROC: receiver operating characteristic. The statistical significance level (α = 0.05) was determined using the t-test.

We used boxplots to validate the expression levels of candidate hub genes. Compared to normal controls, the expressions of CD44 (*p* = 6e-08), ITGB2 (*p* = 4.9e-09), MICB (*p* = 2.9e-14), and RAC2 (*p* = 5.1e-12) were significantly elevated in AAGN glomerular tissue (Figure 7(A)). Subsequently, we validated the expression of these candidate hub genes using the external dataset GSE108109. The results demonstrated that the expression levels of CD44 (*p* = 1.7e-05), ITGB2 (*p* = 0.00011), MICB (*p* = 2.6e-05), and RAC2 (*p* = 0.002) were significantly upregulated in AAGN glomerular tissues (Figure 7(C)). In the AAGN tubulointerstitial compartment, MAPT (*p* = 8.7e-11) and VEGFA (*p* = 5.5e-13) were notably downregulated, while VCAM1 (*p* = 1.3e-10) was significantly upregulated (Figure 7(B)). Further validation in the external dataset GSE108112 revealed that VEGFA (*p* = 0.045) remained significantly downregulated and VCAM1 (*p* = 0.032) was significantly upregulated, whereas MAPT (*p* = 0.12) did not exhibit significant changes (Figure 7(D)). In summary, CD44, ITGB2, MICB, and RAC2 were identified as potential oxidative stress-related hub genes in AAGN glomeruli, while VEGFA and VCAM1 were identified as oxidative stress-related hub genes in the tubulointerstitial compartment.

Subsequently, the diagnostic potential of the hub genes was further evaluated through ROC curve analysis. Hub genes with AUC > 0.7 are regarded as potential diagnostic markers. In the GSE104948 dataset, MICB showed the highest diagnostic potential with an AUC of 1.000 (95% CI: 1.000–1.000). The other hub genes also demonstrated excellent performance, with AUC values of 0.995 (95% CI: 0.983–1.000) for CD44, 0.992 (95% CI: 0.977–1.000) for ITGB2, and 0.987 (95% CI: 0.964–1.000) for RAC2 (Figure 7(E)). Within the tubulointerstitial training set GSE104954, VEGFA achieved a perfect AUC value of 1.000 (95% CI: 1.000–1.000), and VCAM1 also displayed strong diagnostic performance with an AUC of 0.979 (95% CI: 0.946–1.000) (Figure 7(F)). We further validated the diagnostic potential of these hub genes in external validation sets. All four hub genes (CD44, ITGB2, MICB, and RAC2) showed AUC values exceeding 0.9 (Figure 7(G)), indicating excellent diagnostic performance in the glomerular validation set. Additionally, VEGFA and VCAM1 achieved AUC values exceeding 0.7 (Figure 7(H)), indicating their reliable diagnostic potential in the tubulointerstitial validation set.

To comprehensively validate the reliability of our screening results and their tissue-specific expression patterns, we performed expression validation and diagnostic performance evaluation of the four oxidative stress-related hub genes (CD44, ITGB2, MICB, and RAC2) identified from the glomerular dataset using ANCA-associated vasculitis tubulointerstitial datasets (GSE104954 and GSE108112). The analysis revealed that these genes were significantly upregulated in tubulointerstitial tissues (all *p-*values < 0.05) (Supplementary Figure S1(A,B)). Specifically, in the GSE104954 dataset, CD44, ITGB2, MICB, and RAC2 exhibited AUC values of 0.868 (95% CI: 0.744–0.991), 0.886 (95% CI: 0.771–1.000), 0.876 (95% CI: 0.764–0.988), and 0.915 (95% CI: 0.827–1.000), respectively. In the GSE108112 dataset, their AUC values were 0.804 (95% CI: 0.689–0.918), 0.779 (95% CI: 0.614–0.944), 0.877 (95% CI: 0.752–1.000), and 0.758 (95% CI: 0.614–0.902), demonstrating robust diagnostic performance (Supplementary Figure S1(E,F)). To further explore tissue-specific expression patterns, we validated the expression characteristics of the two oxidative stress-related hub genes (VCAM1 and VEGFA) identified from the tubulointerstitial dataset in ANCA-associated vasculitis glomerular tissues (GSE104948 and GSE108109). In the GSE104948 dataset, VCAM1 showed significant upregulation (*p* = 0.00026) with an AUC of 0.814 (95% CI: 0.673–0.956), while VEGFA was significantly downregulated (*p* = 0.002) with an AUC of 0.732 (95% CI: 0.573–0.891) (Supplementary Figure S1(C,G)). However, in the GSE108109 dataset, neither VCAM1 (*p* = 0.58) nor VEGFA (*p* = 0.28) exhibited statistically significant differential expression, with AUC values of 0.589 (95% CI: 0.331–0.847) and 0.589 (95% CI: 0.336–0.842), respectively (Supplementary Figure S1(D,H)).

These results suggest that the oxidative stress-related hub genes CD44, ITGB2, MICB, and RAC2 may play significant roles in both glomerular and tubulointerstitial tissues in ANCA-associated vasculitis, while VCAM1 and VEGFA exhibit distinct tubulointerstitial tissue specificity. This finding provides new insights into the oxidative stress mechanisms in different renal compartments in ANCA-associated vasculitis.

**Figure 8.**
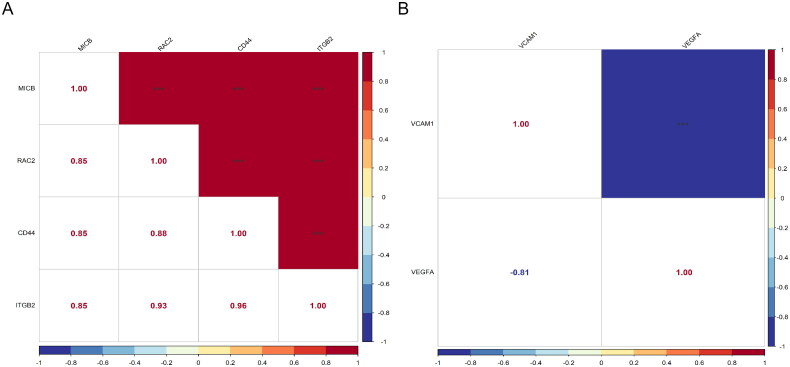
Correlations between expression levels of various hub gene pairs. (A) Correlations between expression levels of various hub gene pairs in the GSE104948 dataset. (B) Correlations between expression levels of various hub gene pairs in the GSE104954 dataset. *p < 0.05, **p < 0.01, *** *p* < 0.001.

Furthermore, we examined the correlations among the hub genes. In the glomerular dataset, all four diagnostic genes displayed positive correlations, with the strongest ­correlation (*r* = 0.96, *p* < 0.001) between CD44 and ITGB2 (Figure 8(A)). In the tubulointerstitial dataset, VEGFA and VCAM1 showed a significant negative correlation (r = −0.81, *p* < 0.001) (Figure 8(B)).

### 3.8. Correlation analysis between hub genes and immune cells

**Figure 9.**
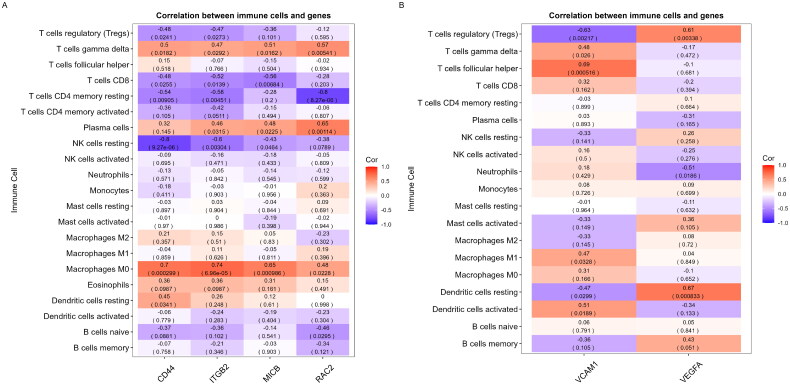
Correlation of hub genes. (A) Correlation between CD44, ITGB2, MICB, and RAC2 with immune infiltrating cells. (B) Correlation between VCAM1 and VEGFA with immune infiltrating cells.

Immune inflammation plays a central role in the development and progression of AAGN. To explore the involvement of these genes in immune infiltration, we performed Spearman correlation analysis to assess their relationships. Correlation analysis involving 22 immune cells and hub genes revealed that M0 macrophages and γδT cells exhibited significant positive correlations with CD44, ITGB2, MICB, and RAC2 in the glomerular dataset (all *p-*values < 0.05) and three of the four hub genes were negatively associated with resting NK cells, T cells CD4 memory resting and T cells CD8 (Figure 9(A)). In the tubulointerstitial dataset, VCAM1 exhibited the most prominent positive correlation with follicular helper T cells (*p* = 0.000516) and the most pronounced negative correlation with Tregs (*p* = 0.00217). VEGFA demonstrated the most notable positive correlation with resting dendritic cells (*p* = 0.000833) and the most marked negative correlation with neutrophils (*p* = 0.0186) (Figure 9(B)). These results further underscore the critical role of these immune cell types in AAGN progression.

### 3.9. GSEA analysis of hub genes

**Figure 10.**
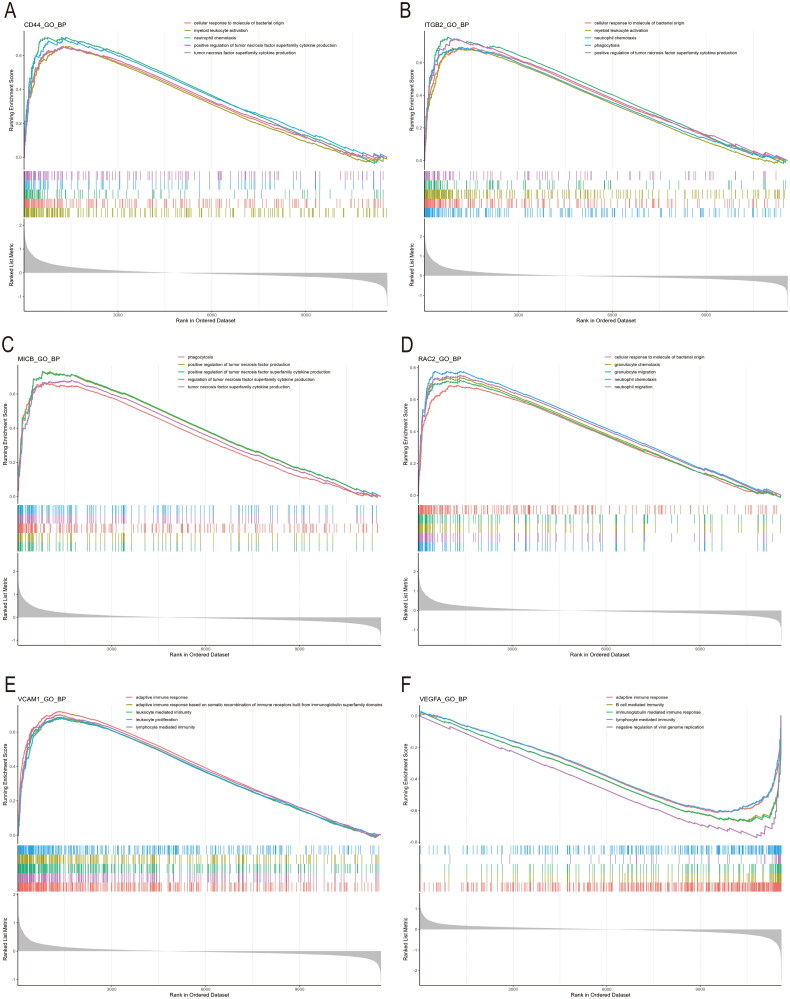
GSEA analysis of hub genes. (A–D) The function of CD44, ITGB2, MICB, and RAC2 using GSEA analysis. (E,F) The function of VCAM1 and VEGFA using GSEA analysis. GSEA: gene set enrichment analysis.

To explore the potential functions of the hub genes, we performed gene set enrichment analysis (GSEA) on individual genes. By comparing samples with high and low expression levels of the candidate genes, we aimed to elucidate their molecular functions and underlying biological pathways. The results revealed that genes in the high-expression cohort of CD44, ITGB2, MICB, and RAC2 within the glomerular compartment dataset were significantly enriched in pathways associated with inflammation and immune response, such as myeloid leukocyte activation, neutrophil chemotaxis, and positive regulation of tumor necrosis factor superfamily cytokine production (Figures 10(A–D)). This suggests that these hub genes may play a role in inflammatory and immune response processes. In the tubulointerstitial dataset, the high-expression cohorts of VCAM1 and VEGFA were similarly enriched in pathways related to inflammation and immunity, including adaptive immune response, B cell-mediated immunity, and immunoglobulin-mediated immunity (Figures 10(E,F)).

### 3.10. Regulatory network construction and potential drug prediction

**Figure 11.**
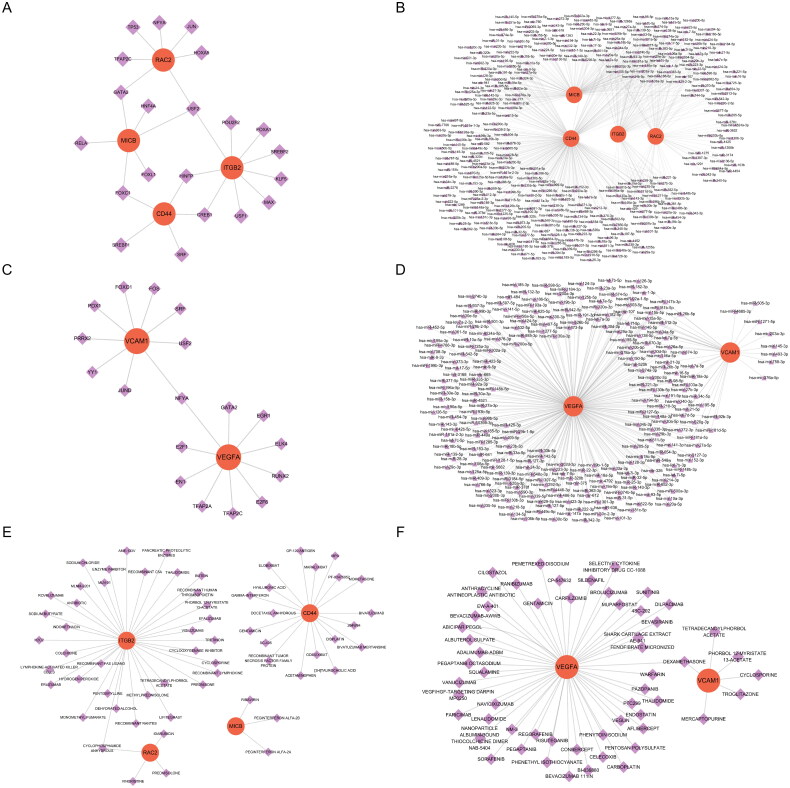
Regulatory networks and target drugs of hub genes. (A) The TF network of CD44, ITGB2, MICB, and RAC2. (B) The miRNA network of CD44, ITGB2, MICB, and RAC2. (C) The TF network of VCAM1 and VEGFA. (D) The miRNA network of VCAM1 and VEGFA. (E) Drug–key genes interaction network of CD44, ITGB2, MICB, and RAC2. (F) Drug–key genes interaction network of VCAM1 and VEGFA. TF: transcription factor.

To predict the transcriptional regulatory effects of transcription factors (TFs) and miRNAs on hub genes, we constructed miRNA-hub gene and TF-hub gene regulatory networks using the NetworkAnalyst database and visualized them using Cytoscape software. In the glomerular dataset, CD44, ITGB2, MICB, and RAC2 interact with 5, 9, 6, and 7 transcription factors respectively; concurrently, these genes are associated with 251, 71, 132, and 62 miRNAs. In the tubulointerstitial dataset, VCAM1 and VEGFA interact with 9 and 10 transcription factors, respectively, and are associated with 35 and 235 miRNAs. Within the glomerular dataset, one TF (USF2) interacts with three hub genes; additionally, twelve miRNAs (e.g., hsa-let-7c-5p, hsa-mir-20a-5p, hsa-mir-34a-5p) engage with four hub genes. In the tubulointerstitial dataset, one TF (NFYA) interacts with two hub genes alongside twenty-seven miRNAs (e.g., hsa-mir-15a-5p, hsa-mir-16-5p, hsa-mir-21-5p), which also regulate these two hub genes. These findings suggest that these miRNAs and transcription factors may have closer interactions with hub genes (Figure 11(A–D)).

To identify potentially effective drugs targeting hub genes, we obtained gene-chemical interaction networks from the Drug-Gene Interaction Database (DGIdb) and visualized them using Cytoscape software. In the glomerular dataset, the four hub genes CD44, ITGB2, MICB, and RAC2 were found to intersect with 19, 36, 3, and 4 drugs/chemicals respectively. Among the 62 drugs identified, cyclophosphamide was associated with two hub genes (RAC2 and ITGB2) (Figure 11(E)). In the tubulointerstitial dataset, two hub genes VCAM1 and VEGFA were associated with 6 and 52 drugs/chemicals respectively; notably, dexamethasone intersected with both of these hub genes (Figure 11(F)).

## 4. Discussion

In this study, we obtained glomerular and tubulointerstitial compartment datasets related to AAGN from the GEO database. By intersecting genes strongly correlated with AAGN immune cell infiltration, as identified by WGCNA, we derived the differentially expressed immune oxidative stress-related genes (DEIOSGs). The glomerular DEIOSGs were primarily enriched in GO terms such as oxidative stress response, regulation of inflammatory response, and leukocyte migration. In contrast, the DEIOSGs in the tubulointerstitial compartments were mainly associated with oxidative stress response, response to exogenous stimuli, and response to oxygen levels. KEGG pathway analysis revealed significant enrichment of DEIOSGs in pathways related to lipid atherosclerosis and AGE-RAGE signaling pathways in diabetic complications within both glomerular and tubulointerstitial compartments. Our functional enrichment analysis revealed that DEIOSGs are predominantly involved in immunoinflammatory and oxidative stress-related pathways. These results emphasize the importance of immunoinflammation and oxidative stress in AAGN, suggesting that therapeutic approaches focused on oxidative stress hold significant promise.

We identified four hub genes related to oxidative stress in AAGN glomeruli-CD44, ITGB2, MICB, and RAC2 – all of which were significantly upregulated. In the tubulointerstitial compartments, two oxidative stress-related hub genes, VCAM1 and VEGFA, were identified. Notably, VCAM1 was significantly upregulated in AAV patients, while VEGFA was significantly downregulated. ROC curve analyses also produced significant results, with AUC values for CD44, ITGB2, MICB, and RAC2 surpassing 0.9 and VCAM1 and VEGFA achieving AUC values greater than 0.7. These results suggest that these oxidative stress-related hub genes hold considerable diagnostic value as potential biomarkers for AAGN. The hub genes identified through this integrative approach may play a critical role in unraveling the pathogenesis of AAV-related kidney injury.

As a member of the Cell Adhesion Molecule (CAM) family, CD44 plays an important role in signal transduction and cell adhesion between cells and extracellular matrix and is involved in many physiological processes such as T lymphocyte activation, tissue remodeling, and cell migration [24]. Previous studies have demonstrated that CD44 expression is upregulated in activated monocyte-derived macrophages (AMDM). This upregulation enhances the copper uptake capacity of these cells, facilitates hydrogen peroxide activation to catalyze the NAD(H) redox cycle, and consequently plays a role in regulating inflammation and oxidative stress [25]. The loss of CD44 results in decreased activation and proliferation of glomerular parietal epithelial cells (PECs), subsequently reducing both crescent formation and proteinuria [26,27]. MICB is part of the MHC locus on human chromosome 6, and MIC protein is regarded as a marker of ‘stress’ in epithelial cells. MICB is also an immune-activating ligand of the killer cell lectin-like receptor K1 (KLRK1)/NKG2D receptor and can be recognized as a stress-induced autoantigen by γδT cells [28–30]. Currently, no studies have elucidated the relationship between MICB and AAGN, but elevated levels of MICB expression are significantly correlated with impaired renal function, including a reduced glomerular filtration rate (GFR) and increased serum creatinine levels [31]. ITGB2, a member of the integrin family, is predominantly expressed in immune cells and is intricately associated with various immune functions, including leukocyte extravasation, complement fragment binding, and intracellular killing of pathogenic microorganisms. ITGB2 is also implicated in mediating responses to oxidative stress. Research by Zhang X et al. demonstrated that oxidative stress promotes the proliferation of cancer-associated fibroblasts (CAFs) by impairing mitochondrial functions (such as NADH oxidation). Although the research primarily focused on CAFs within tumor microenvironments, ITGB2, as an integrin family member, may similarly regulate oxidative stress responses in immune cells, highlighting its potential broader functional significance [32,33]. RAC2, a member of the Rho subfamily of RAS-related guanosine triphosphatases, can transition from an inactive GDP-bound state to an active GTP-bound state upon receptor stimulation. The GTP-bound form of RAC2 is crucial for activating the NADPH oxidase complex, which drives superoxide production and plays a pivotal role in oxidative stress responses [34,35]. However, current research on the relationship between ITGB2, RAC2, and kidney disease remains inadequate. In our study, we observed that upregulated levels of ITGB2 and RAC2 exhibited superior diagnostic efficacy (AUC > 0.90). Although their molecular mechanisms are not yet fully elucidated, we propose this as the first identification of them as biomarkers for AAGN, and it’s necessary to further explore their roles in the mechanism.

VEGFA, a secreted protein, stimulates angiogenesis in hypoxic conditions and supports the survival of renal epithelial cells. The PI3K/Akt pathway can increase VEGF transcription, while decreased VEGFA levels may lead to reduced endothelial cell survival and impaired angiogenesis [36,37]. Previous research has reported low VEGFA expression in the renal tubulointerstitial compartment of diabetic nephropathy (DN) patients [38]. Similarly, our study also found downregulated VEGFA expression in the tubulointerstitial compartments of AAGN patients. VCAM1 is a protein expressed on vascular endothelial cells that plays a critical role in inflammation and vascular disease. Previous studies have shown that in the necrotic areas of glomerular capillary loops related to ANCA-related crescentic nephritis [39,40], VCAM1 expression is significantly elevated, along with extensive inflammatory cell infiltration. Seron et al. examined VCAM1 expression in renal tissues from 50 patients with glomerular and tubulointerstitial diseases. They found varying degrees of increased VCAM1 expression in renal tubular epithelial cells of patients with crescentic nephritis, vasculitis, lupus nephritis, and IgA nephropathy. Notably, the increase was most pronounced in patients with vasculitis [41]. It is suggested that abnormal VCAM1 expression in these necrotic areas could serve as a potential marker for ANCA-associated necrotizing glomerulosclerosis.

In addition, we constructed gene regulatory networks and investigated potential molecular-targeting drugs for AAGN. TFs are crucial protein complexes that regulate gene transcription by recognizing specific DNA sequences. MiRNAs are small non-coding RNA molecules that inhibit translation by binding to one or more sites on mRNA transcripts or participate in post-transcriptional regulation through TF modulation, significantly impacting various cellular and biological processes. Our analysis revealed distinct regulatory networks in glomerular and tubulointerstitial datasets. In the glomerular dataset, the transcription factor USF2 was found to interact with three hub genes, while a network of 12 miRNAs (including hsa-let-7c-5p, hsa-mir-20a-5p, and hsa-mir-34a-5p) demonstrated interactions with four hub genes. The tubulointerstitial dataset showed more extensive regulatory interactions, with transcription factor NFYA and 27 miRNAs (including hsa-mir-15a-5p, hsa-mir-16-5p, and hsa-mir-21-5p) co-regulating both VCAM1 and VEGFA, suggesting their potential synergistic role in modulating these targets. Furthermore, our drug-target analysis identified specific therapeutic agents that interact with these hub genes: cyclophosphamide was predicted to target RAC2 and ITGB2, whereas dexamethasone showed potential interactions with VCAM1 and VEGFA.

Cyclophosphamide, a widely used alkylating chemotherapeutic agent, exerts its immunosuppressive effects by inhibiting the proliferation of immune cells, particularly T cells and B cells, making it a common treatment for autoimmune diseases and transplant rejection [42]. *In vivo*, cyclophosphamide is metabolized in the liver into its active forms, such as phosphoramide mustard. These metabolites form covalent cross-links with DNA, blocking DNA replication and transcription, thereby suppressing the proliferation and activation of immune cells, such as T cells, B cells, and neutrophils [43]. This process may indirectly influence the activity of RAC2 and ITGB2. Additionally, cyclophosphamide may protect cells from oxidative stress through the Nrf2-mediated antioxidant pathway [44], potentially modulating signaling pathways associated with RAC2 and ITGB2. Although the precise mechanisms by which cyclophosphamide affects RAC2 and ITGB2 remain unclear, we hypothesize that its immunomodulatory and oxidative stress-related effects may regulate their functions. Further studies are needed to elucidate these interactions.

Dexamethasone, a potent glucocorticoid, exhibits diverse biological effects, including anti-inflammatory, immunosuppressive, and anti-angiogenic properties. Research has shown that dexamethasone markedly decreases VCAM1 expression, primarily by inhibiting NF-κB activation and suppressing the release of pro-inflammatory cytokines such as IL-6 and TNF-α [45]. These actions contribute to the direct and indirect downregulation of VCAM1, highlighting dexamethasone’s potent anti-inflammatory effects. Furthermore, dexamethasone directly inhibits VEGFA expression. By suppressing HIF-1α, a key regulator of VEGFA under hypoxic conditions, dexamethasone significantly lowers VEGFA levels [46], highlighting its broad therapeutic potential in modulating both inflammation and angiogenesis.

Our study provides novel insights into immune-associated oxidative stress genes in the glomerular and tubulointerstitial compartments of AAGN, but it is not without limitations. First, the evidence relies on publicly available data; while we conducted expression validation using independent external datasets, additional prospective studies and further *in vitro* and *in vivo* experiments are necessary to confirm these results before the clinical application of the diagnostic markers can be considered. Second, the sample size of the sequencing dataset is relatively small, necessitating improvements in this area. Third, reliance on retrospective data from public repositories may introduce bias and limit the generalizability of our findings.

## 5. Conclusion

In conclusion, this study significantly enhances our understanding of oxidative stress in the glomerular and tubulointerstitial compartments of AAGN by employing comprehensive bioinformatics strategies. We identified four oxidative stress-related hub genes (CD44, ITGB2, MICB, and RAC2) in the glomerular compartment and two (VCAM1 and VEGFA) in the tubulointerstitial compartment. These findings suggest that these hub genes may play critical roles in driving AAGN progression, potentially through immune and inflammatory mechanisms.

## Supplementary Material

Supplementary material.docx

Supplementary FigureS1.tif

## Data Availability

Datasets (GSE104948, GSE104954, GSE108109, and GSE108112) used in this study are available in the Gene Expression Omnibus (GEO) repository (https://www.ncbi.nlm.nih.gov/geo/), further inquiries can be directed to the corresponding authors.

## References

[1] Jennette JC, Nachman PH. ANCA glomerulonephritis and vasculitis. Clin J Am Soc Nephrol. 2017;12(10):1680–1691. doi: 10.2215/CJN.02500317. 28842398 PMC5628710

[2] Geetha D, Jefferson JA. ANCA-associated vasculitis: core curriculum 2020. Am J Kidney Dis. 2020;75(1):124–137. doi: 10.1053/j.ajkd.2019.04.031. 31358311

[3] Jennette JC, Falk RJ, Bacon PA, et al. 2012 revised International Chapel Hill Consensus Conference nomenclature of vasculitides. Arthritis Rheum. 2013;65(1):1–11. doi: 10.1002/art.37715. 23045170

[4] Kronbichler A, Lee KH, Denicolò S, et al. Immunopathogenesis of ANCA-associated vasculitis. Int J Mol Sci. 2020;21(19):7319. doi: 10.3390/ijms21197319. 33023023 PMC7584042

[5] Amsler J, Everts-Graber J, Martin KR, et al. Dysregulation of neutrophil oxidant production and interleukin-1-­related cytokines in granulomatosis with polyangiitis. Rheumatology (Oxford). 2024;63(8):2249–2258. doi: 10.1093/rheumatology/kead578. 37947315

[6] Shimojima Y, Kishida D, Ichikawa T, et al. Oxidative stress promotes instability of regulatory T cells in antineutrophil cytoplasmic antibody-associated vasculitis. Front Immunol. 2021;12:789740. doi: 10.3389/fimmu.2021.789740. 34950150 PMC8691772

[7] Wu L, Wang G, Yang B, et al. Urinary matrix metalloproteinase 7 activated by oxidative stress predicts kidney prognosis in myeloperoxidase-antineutrophil cytoplasmic antibody-associated vasculitis. Antioxid Redox Signal. 2022;37(4–6):246–256. doi: 10.1089/ars.2021.0188. 35152729

[8] Xiao H, Hu P, Falk RJ, et al. Overview of the pathogenesis of ANCA-associated vasculitis. Kidney Diseases (Basel, Switzerland). 2016;1(4):205–215. 27536680 10.1159/000442323 PMC4934824

[9] Shochet L, Holdsworth S, Kitching AR. Animal models of ANCA associated vasculitis. Front Immunol. 2020;11:525. doi: 10.3389/fimmu.2020.00525. 32373109 PMC7179669

[10] Kimura H, Matsuyama Y, Araki S, et al. The effect and ­possible clinical efficacy of in vivo inhibition of neutrophil extracellular traps by blockade of PI3K-gamma on the pathogenesis of microscopic polyangiitis. Mod Rheumatol. 2018;28(3):530–541. doi: 10.1080/14397595.2017.1367116. 28880680

[11] Khan MI, Dębski KJ, Dabrowski M, et al. Gene set enrichment analysis and ingenuity pathway analysis of metastatic clear cell renal cell carcinoma cell line. Am J Physiol Renal Physiol. 2016;311(2):F424–436. doi: 10.1152/ajprenal.00138.2016. 27279483

[12] Barrett T, Wilhite SE, Ledoux P, et al. NCBI GEO: archive for functional genomics data sets–update. Nucleic Acids Res. 2013;41(Database issue):D991–995. doi: 10.1093/nar/gks1193. 23193258 PMC3531084

[13] Sui S, An X, Xu C, et al. An immune cell infiltration-based immune score model predicts prognosis and ­chemotherapy effects in breast cancer. Theranostics. 2020;10(26):11938–11949. doi: 10.7150/thno.49451. 33204321 PMC7667685

[14] Langfelder P, Horvath S. WGCNA: an R package for weighted correlation network analysis. BMC Bioinformat. 2008;9(1):559. doi: 10.1186/1471-2105-9-559. PMC2631488 19114008

[15] Ashburner M, Ball CA, Blake JA, et al. Gene ontology: tool for the unification of biology. The Gene Ontology Consortium. Nat Genet. 2000;25(1):25–29. doi: 10.1038/75556. 10802651 PMC3037419

[16] Kanehisa M, Goto S. KEGG: kyoto encyclopedia of genes and genomes. Nucleic Acids Res. 2000;28(1):27–30. doi: 10.1093/nar/28.1.27. 10592173 PMC102409

[17] Duan J, Soussen C, Brie D, et al. Generalized LASSO with under-determined regularization matrices. Signal Process. 2016;127:239–246. doi: 10.1016/j.sigpro.2016.03.001. PMC4917299 27346902

[18] Lin X, Li C, Zhang Y, et al. Selecting feature subsets based on SVM-RFE and the overlapping ratio with applications in bioinformatics. Molecules. 2017;23(1):52. doi: 10.3390/molecules23010052. 29278382 PMC5943966

[19] Uddin S, Khan A, Hossain ME, et al. Comparing different supervised machine learning algorithms for disease prediction. BMC Med Inform Decis Mak. 2019;19(1):281. doi: 10.1186/s12911-019-1004-8. 31864346 PMC6925840

[20] Robin X, Turck N, Hainard A, et al. pROC: an open-source package for R and S + to analyze and compare ROC curves. BMC Bioinformat. 2011;12(1):77. doi: 10.1186/1471-2105-12-77. PMC3068975 21414208

[21] Wu T, Hu E, Xu S, et al. clusterProfiler 4.0: a universal enrichment tool for interpreting omics data. Innovation (Camb). 2021;2(3):100141. doi: 10.1016/j.xinn.2021.100141. 34557778 PMC8454663

[22] Zhou G, Soufan O, Ewald J, et al. NetworkAnalyst 3.0: a visual analytics platform for comprehensive gene expression profiling and meta-analysis. Nucleic Acids Res. 2019;47(W1):W234–w241. doi: 10.1093/nar/gkz240. 30931480 PMC6602507

[23] Freshour SL, Kiwala S, Cotto KC, et al. Integration of the Drug-Gene Interaction Database (DGIdb 4.0) with open crowdsource efforts. Nucleic Acids Res. 2021;49(D1):D1144–d1151. doi: 10.1093/nar/gkaa1084. 33237278 PMC7778926

[24] Funaro A, Spagnoli GC, Momo M, et al. Stimulation of T cells via CD44 requires leukocyte-function-associated antigen interactions and interleukin-2 production. Hum Immunol. 1994;40(4):267–278. doi: 10.1016/0198-8859(94)90026-4. 7528188

[25] Solier S, Müller S, Cañeque T, et al. A druggable copper-signalling pathway that drives inflammation. Nature. 2023;617(7960):386–394. doi: 10.1038/s41586-023-06017-4. 37100912 PMC10131557

[26] Anguiano L, Kain R, Anders HJ. The glomerular crescent: triggers, evolution, resolution, and implications for therapy. Curr Opin Nephrol Hypertens. 2020;29(3):302–309. doi: 10.1097/MNH.0000000000000596. 32132388 PMC7170443

[27] Eymael J, Sharma S, Loeven MA, et al. CD44 is required for the pathogenesis of experimental crescentic glomerulonephritis and collapsing focal segmental glomerulosclerosis. Kidney Int. 2018;93(3):626–642. doi: 10.1016/j.kint.2017.09.020. 29276101

[28] Sutherland CL, Chalupny NJ, Schooley K, et al. UL16-binding proteins, novel MHC class I-related proteins, bind to NKG2D and activate multiple signaling pathways in ­primary NK cells. J Immunol. 2002;168(2):671–679. doi: 10.4049/jimmunol.168.2.671. 11777960

[29] Steinle A, Li P, Morris DL, et al. Interactions of human NKG2D with its ligands MICA, MICB, and homologs of the mouse RAE-1 protein family. Immunogenetics. 2001;53(4):279–287. doi: 10.1007/s002510100325. 11491531

[30] Groh V, Steinle A, Bauer S, et al. Recognition of stress-induced MHC molecules by intestinal epithelial gammadelta T cells. Science. 1998;279(5357):1737–1740. doi: 10.1126/science.279.5357.1737. 9497295

[31] Fan C, Gao Y, Sun Y. Integrated multiple-microarray analysis and mendelian randomization to identify novel targets involved in diabetic nephropathy. Front Endocrinol (Lausanne). 2023;14:1191768. doi: 10.3389/fendo.2023.1191768. 37492198 PMC10363738

[32] Zhang X, Dong Y, Zhao M, et al. ITGB2-mediated metabolic switch in CAFs promotes OSCC proliferation by oxidation of NADH in mitochondrial oxidative phosphorylation system. Theranostics. 2020;10(26):12044–12059. doi: 10.7150/thno.47901. 33204328 PMC7667693

[33] Tan SM. The leucocyte β2 (CD18) integrins: the structure, functional regulation and signalling properties. Biosci Rep. 2012;32(3):241–269. doi: 10.1042/BSR20110101. 22458844

[34] Zou Y, Xiong JB, Ma K, et al. Rac2 deficiency attenuates CCl(4)-induced liver injury through suppressing inflammation and oxidative stress. Biomed Pharmacother. 2017;94:140–149. doi: 10.1016/j.biopha.2017.07.074. 28759751

[35] Xu L, Ren H, Xie D, et al. Rac2 mediate foam cell formation and associated immune responses in THP-1 to ­promote the process of atherosclerotic plaques. Mol Immunol. 2023;163:196–206. doi: 10.1016/j.molimm.2023.10.004. 37837955

[36] Villegas G, Lange-Sperandio B, Tufro A. Autocrine and paracrine functions of vascular endothelial growth factor (VEGF) in renal tubular epithelial cells. Kidney Int. 2005;67(2):449–457. doi: 10.1111/j.1523-1755.2005.67101.x. 15673292

[37] Sun B, Lu C, Zhou GP, et al. Suppression of Par-4 protects human renal proximal tubule cells from apoptosis induced by oxidative stress. Nephron Exp Nephrol. 2011;117(3):e53-61–e61. doi: 10.1159/000320594. 20814219

[38] Lindenmeyer MT, Kretzler M, Boucherot A, et al. Interstitial vascular rarefaction and reduced VEGF-A expression in ­human diabetic nephropathy. J Am Soc Nephrol. 2007;18(6):1765–1776. doi: 10.1681/ASN.2006121304. 17475821

[39] Rastaldi MP, Ferrario F, Tunesi S, et al. Intraglomerular and interstitial leukocyte infiltration, adhesion molecules, and interleukin-1 alpha expression in 15 cases of antineutrophil cytoplasmic autoantibody-associated renal vasculitis. Am J Kidney Dis. 1996;27(1):48–57. doi: 10.1016/s0272-6386(96)90030-x. 8546138

[40] D’Amico G, Sinico RA, Ferrario F. Renal vasculitis. Nephrol Dial Transplant. 1996;11 Suppl 9:69–74. doi: 10.1093/ndt/11.supp9.69. 9050038

[41] Seron D, Cameron JS, Haskard DO. Expression of VCAM-1 in the normal and diseased kidney. Nephrol Dial Transplant. 1991;6(12):917–922. doi: 10.1093/ndt/6.12.917. 1724689

[42] Ahlmann M, Hempel G. The effect of cyclophosphamide on the immune system: implications for clinical cancer therapy. Cancer Chemother Pharmacol. 2016;78(4):661–671. doi: 10.1007/s00280-016-3152-1. 27646791

[43] Ibrahim KM, Darwish SF, Mantawy EM, et al. Molecular mechanisms underlying cyclophosphamide-induced cognitive impairment and strategies for neuroprotection in preclinical models. Mol Cell Biochem. 2024;479(8):1873–1893. doi: 10.1007/s11010-023-04805-0. 37522975 PMC11339103

[44] Ni B, Chen Z, Shu L, et al. Nrf2 pathway ameliorates bladder dysfunction in cyclophosphamide-induced cystitis via suppression of oxidative stress. Oxid Med Cell Longev. 2021;2021(1):4009308. doi: 10.1155/2021/4009308. 34306306 PMC8279868

[45] Vykhovanets EV, Shukla S, MacLennan GT, et al. Il-1 beta-induced post-transition effect of NF-kappaB provides time-dependent wave of signals for initial phase of intrapostatic inflammation. Prostate. 2009;69(6):633–643. doi: 10.1002/pros.20916. 19170127 PMC2669895

[46] Xiao B, Wang S, Yang G, et al. HIF-1α contributes to hypoxia adaptation of the naked mole rat. Oncotarget. 2017;8(66):109941–109951. doi: 10.18632/oncotarget.22767. 29299120 PMC5746355

