# Supplementary material for: Unveiling the role of oxidative stress in ANCA-associated glomerulonephritis through integrated machine learning and bioinformatics analyses
Source: Ren Fail. 2025 May 14;47(1):2499905. doi: 10.1080/0886022X.2025.2499905 (PMC12082741; doi:10.1080/0886022X.2025.2499905)
Supplement: Supplementary material.docx [file IRNF_A_2499905_SM2732.docx]

Supplementary Figure S1. Expression of hub genes. (A). Expression of hub genes (VCAM1 and VEGFA) in the GSE104948 dataset. (B). Expression of hub genes (VCAM1 and VEGFA) in the GSE108109 dataset. (C). Expression of hub genes (CD44, ITGB2, MICB, and RAC2) in the GSE104954 dataset. (C). Expression of hub genes (CD44, ITGB2, MICB, and RAC2) in the GSE108112 dataset. ANCA: anti-neutrophil cytoplasmic antibody; LD: living donors

Supplementary Table S1. DEIOSGs from the GSE104948 and GSE104954 datasets.

| GSE104948 | |  | GSE104954 | |
| --- | --- | --- | --- | --- |
| IGF1 | CXCL8 |  | KL | CASP4 |
| NR3C2 | TIMP1 |  | HADH | COL1A1 |
| PC | MMP1 |  | DLST | TPM1 |
| ACOX2 | AREG |  | CYP2B6 | IRF1 |
| HYAL1 | MICB |  | HDAC6 | PDLIM1 |
| PDK2 | ADA |  | RORA | CXCL1 |
| PRKCZ | MELK |  | ALAD | PON2 |
| RORA | LYN |  | ACAA1 | SELL |
| MAPT | CCL2 |  | VDR | UCP2 |
| RCAN1 | CXCR4 |  | CYP3A4 | LYN |
| PIK3CG | SOD2 |  | EPHX2 | TLR2 |
| BAX | PLA2G7 |  | PDK2 | DNMT1 |
| TUBB3 | ALOX5 |  | FMO5 | ITGAM |
| PRR5L | CD44 |  | VEGFA | HIF1A |
| CCNA2 | UCP2 |  | ACOX2 | BCL2A1 |
| GLRX2 | TNFRSF1B |  | ASS1 | CXCR4 |
| CASP4 | OLR1 |  | NET1 | CD44 |
| PPARG | SERPINE1 |  | PAH | AKR1B1 |
| LGALS3 | BCL2A1 |  | CYP4A11 | FAS |
| NOD2 | COL1A1 |  | FABP1 | ECT2 |
| MCTP1 | RAC2 |  | ALDH3A2 | ADAM9 |
| DSP | IGFBP1 |  | KNG1 | GSTP1 |
| HBEGF | CXCL1 |  | MAPT | ME1 |
| SLC1A3 | MMP9 |  | PLG | HSP90B1 |
| MANF | ITGAM |  | GSTA1 | MUC1 |
| BID | CCL4 |  | EGF | CCL4 |
| CDK1 | FN1 |  | CYP3A7 | ARL6IP5 |
| ECT2 | TLR2 |  | APOH | SPP1 |
| CDKN3 | CYBB |  | LTF | ANXA5 |
| PDLIM4 | ITGB2 |  | TIMP1 | TOP2A |
| CYBA | NCF2 |  | SLPI | FN1 |
| PTPN6 | CD36 |  | LCN2 | TGM2 |
| PLAU |  |  | VCAM1 | CD38 |
| IL1B |  |  | CLU | TUBA1B |
| PLA2G4A |  |  | SOD2 | STK39 |
| TREM2 |  |  | VIM | LBR |
| CLEC4A |  |  | ANXA2 | MELK |
| SCO2 |  |  | CCL5 | CASP3 |
| ADRB2 |  |  | HLA-B |  |
| BTK |  |  | ITGB2 |  |
| MYC |  |  | PXDN |  |
| NCF4 |  |  | STAT1 |  |
| HMOX1 |  |  | TNFSF10 |  |
| SPHK1 |  |  | CASP1 |  |
| KCNJ2 |  |  | ALOX5 |  |
| MMP3 |  |  | MYC |  |
| EZH2 |  |  | CYBB |  |
| SLPI |  |  | S100A9 |  |
| CASP1 |  |  | NCF2 |  |
| TOP2A |  |  | RAC2 |  |

DEIOSGs: differentially expressed immune-related oxidative stress genes

Supplementary Table S2. Genelist identified by machine learning.

| GSE104948 | | |  | GSE104954 | | |
| --- | --- | --- | --- | --- | --- | --- |
| LASSO | SVM-RFE | RF |  | LASSO | SVM-RFE | RF |
| IGF1 | ADA | ADA |  | DLST | VEGFA | TGM2 |
| PDK2 | CYBB | MICB |  | VEGFA | MAPT | VEGFA |
| PRKCZ | NR3C2 | PDK2 |  | MAPT | TGM2 | LTF |
| RCAN1 | PRR5L | CYBB |  | LTF |  | MAPT |
| PPARG | PPARG | TIMP1 |  | TNFSF10 |  | ACAA1 |
| TIMP1 | SPHK1 | CASP4 |  |  |  | CYP3A7 |
| MICB | MICB | MANF |  |  |  | SPP1 |
| RAC2 | NOD2 | PRR5L |  |  |  | CASP3 |
| CXCL1 | RAC2 | RAC2 |  |  |  | APOH |
|  | CCL4 | CD44 |  |  |  | VCAM1 |
|  | CXCL1 | GLRX2 |  |  |  | HDAC6 |
|  | ADRB2 | NOD2 |  |  |  | CYP3A4 |
|  | ITGB2 | BTK |  |  |  | DNMT1 |
|  | MAPT | PTPN6 |  |  |  | ANXA5 |
|  | BAX | ITGB2 |  |  |  | SOD2 |
|  | TNFRSF1B | SOD2 |  |  |  | PDK2 |
|  | BID | CD36 |  |  |  | DLST |
|  | GLRX2 | CASP1 |  |  |  | VDR |
|  | CD36 | ALOX5 |  |  |  | TPM1 |
|  | MANF | PIK3CG |  |  |  | ASS1 |
|  | PRKCZ |  |  |  |  |  |
|  | CASP4 |  |  |  |  |  |

LASSO: least absolute shrinkage and selection operator; RF: random forest; SVM-RFE: support vector machine recursive feature elimination
