# Supplementary figures and images for: Unveiling the role of oxidative stress in ANCA-associated glomerulonephritis through integrated machine learning and bioinformatics analyses
Source: Ren Fail. 2025 May 14;47(1):2499905. doi: 10.1080/0886022X.2025.2499905 (PMC12082741; doi:10.1080/0886022X.2025.2499905)

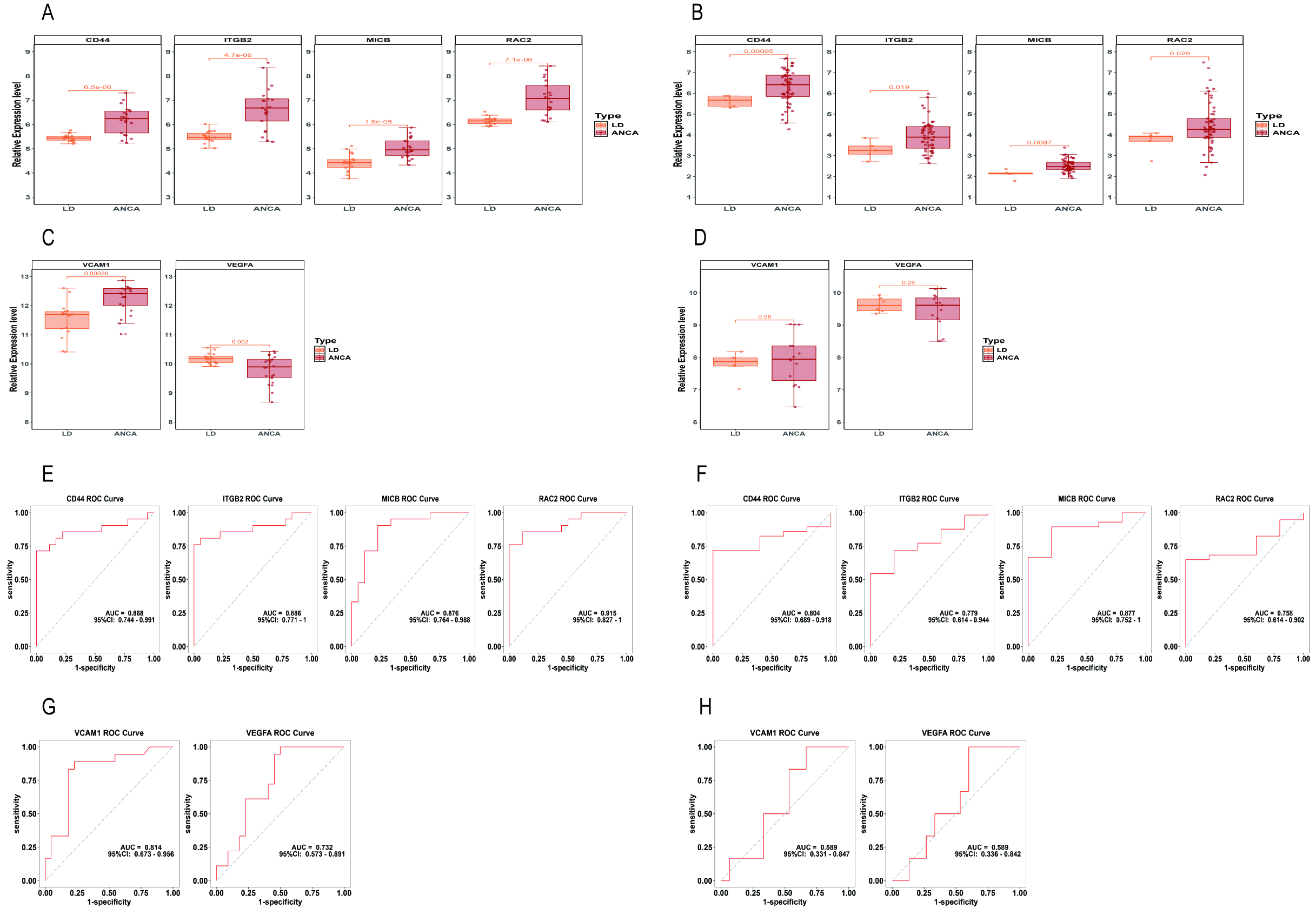

Supplement: Supplementary FigureS1.tif [file IRNF_A_2499905_SM2731.tif]
